# A Hyper-Glycosylation of HBV Surface Antigen Correlates with HBsAg-Negativity at Immunosuppression-Driven HBV Reactivation in Vivo and Hinders HBsAg Recognition In Vitro

**DOI:** 10.3390/v12020251

**Published:** 2020-02-23

**Authors:** Romina Salpini, Lorenzo Piermatteo, Arianna Battisti, Luna Colagrossi, Marianna Aragri, Katia Yu La Rosa, Ada Bertoli, Patrizia Saccomandi, Miriam Lichtner, Massimo Marignani, Sarah Maylin, Constance Delaugerre, Filomena Morisco, Nicola Coppola, Aldo Marrone, Nerio Iapadre, Carlotta Cerva, Stefano Aquaro, Mario Angelico, Loredana Sarmati, Massimo Andreoni, Jens Verheyen, Francesca Ceccherini-Silberstein, Massimo Levrero, Carlo Federico Perno, Laura Belloni, Valentina Svicher

**Affiliations:** 1 Department of Experimental Medicine, University of Rome Tor Vergata, 00133 Rome, Italy; r.salpini@gmail.com (R.S.); piermatteolorenzo@gmail.com (L.P.); battisti.arianna@gmail.com (A.B.); luna_colagrossi@unimi.it (L.C.); marianna.aragri@gmail.com (M.A.); y.katia@hotmail.it (K.Y.L.R.); bertoli@uniroma2.it (A.B.); patrizia.saccomandi@uniroma.2 (P.S.); ceccherini@med.uniroma2.it (F.C.-S.); 2 Public Health and Infectious Disease Department, Sapienza University, 00185 Rome, Italy; miriam.lichtner@gmail.com; 3 Department of Gastroenterology, S.Andrea Hospital, 00189 Rome, Italy; mmarignani@hotmail.com; 4 Laboratoire de Virologie, AP-HP Hopital Saint-Louis, 75010 Paris, France; sarah.maylin@sls.aphp.fr (S.M.); constance.delaugerre@sls.aphp.fr (C.D.); 5 Department of Clinical Medicine and Surgery, Section of Infectious Diseases, University of Naples Federico II, 80138 Naples, Italy; filomena.morisco@unina.it; 6 Department of Mental Health and Public Medicine, Section of Infectious Diseases, Second University of Naples, 80138 Naples, Italy; nicola.coppola@unicampania.it; 7 Internal Medicine and Hepatology Unit, Second University of Naples, 80138 Naples, Italy; aldo.marrone@unicampania.it; 8 Infectious Diseases Unit, San Salvatore Hospital, 67100 L’Aquila, Italy; nerioiapadre@libero.it; 9 Infectious Diseases Unit, Tor Vergata University Hospital, 00133 Rome, Italy; carlottacerva@gmail.com (C.C.); sarmati@med.uniroma2.it (L.S.); andreoni@uniroma2.it (M.A.); 10 Department of Pharmacy, Health and Nutritional Sciences, University of Calabria, 87036 Rende, Italy; aquaro@uniroma2.it; 11 Hepatology Unit, Tor Vergata University Hospital, 00133 Rome, Italy; angelico@med.uniroma2.it; 12 Institute of Virology, University-Hospital, University Duisburg-Essen, 47057 Essen, Germany; jens.verheyen@uk-essen.de; 13 Department of Internal Medicine-DMISM, Sapienza University, 00185 Rome, Italy; massimo.levrero@gmail.com (M.L.); laura.belloni@gmail.com (L.B.); 14 INSERM U1052-Cancer Research Center of Lyon (CRCL), University of Lyon, UMR_S1052, 69008 Lyon, France; 15 Department of Oncology and Haemato-oncology, University of Milan, 20122 Milan, Italy; cf.perno@uniroma2.it; 16 Center for Life NanoSciences (CLNS), IIT-Sapienza, 00133 Rome, Italy

**Keywords:** HBV, HBV reactivation, HBsAg, N-linked glycosylation

## Abstract

Immune-suppression driven Hepatitis B Virus (HBV)-reactivation poses serious concerns since it occurs in several clinical settings and can result in severe forms of hepatitis. Previous studies showed that HBV strains, circulating in patients with HBV-reactivation, are characterized by an enrichment of immune-escape mutations in HBV surface antigen (HBsAg). Here, we focused on specific immune-escape mutations associated with the acquisition of N-linked glycosylation sites in HBsAg (NLGSs). In particular, we investigated profiles of NLGSs in 47 patients with immunosuppression-driven HBV-reactivation and we evaluated their impact on HBsAg-antigenicity and HBV-replication in vitro. At HBV-reactivation, despite a median serum HBV-DNA of 6.7 [5.3–8.0] logIU/mL, 23.4% of patients remained HBsAg-negative. HBsAg-negativity at HBV-reactivation correlated with the presence of >1 additional NLGSs (*p* < 0.001). These NLGSs are located in the major hydrophilic region of HBsAg (known to be the target of antibodies) and resulted from the single mutation T115N, T117N, T123N, N114ins, and from the triple mutant S113N+T131N+M133T. In vitro, NLGSs strongly alter HBsAg antigenic properties and recognition by antibodies used in assays for HBsAg-quantification without affecting HBsAg-secretion and other parameters of HBV-replication. In conclusion, additional NLGSs correlate with HBsAg-negativity despite HBV-reactivation, and hamper HBsAg-antigenicity in vitro, supporting the role of NGSs in immune-escape and the importance of HBV-DNA for a proper diagnosis of HBV-reactivation.

## 1. Introduction

Hepatitis B virus (HBV) reactivation is defined as the abrupt reappearance of HBV in the serum of a person with a previously resolved infection or a marked increase of HBV in an immunosuppressed patient with a previously stable chronic infection [1,2,3]. HBV reactivation occurs in a wide range of clinical settings characterized by different degrees of immunosuppression. The risk of HBV reactivation is related to the establishment of the HBV-DNA minichromosome named covalently closed circular DNA (cccDNA) in hepatocytes during the early phases of HBV infection and to its persistence for a long time (potentially lifelong) after the clinical resolution of infection. The transcriptional activity of cccDNA (template for viral transcripts and crucial for replication activity) is strictly controlled by the epigenetic status of the viral minichromosome that in turn is heavily influenced by signals from the innate and adaptive arms of the immune system [4]. Under immunosuppressive conditions, an impairment of this control can allow cccDNA to enhance its activity and to start the reactivation phase.

HBV reactivation can be clinically severe, resulting in death for acute liver failure or progressive liver disease and cirrhosis [1].

Virological factors associated with immunosuppression-driven HBV reactivation have not been comprehensively clarified yet but immune-escape mutations in the major hydrophilic region (MHR) of HBV surface antigen (HBsAg) seem to be involved in mechanisms underlying HBV reactivation [5,6].

In our previous studies, we have analyzed the genetic characteristics of HBV variants in patients experiencing immunosuppression-driven HBV reactivation, highlighting the role of HBsAg genetic variability in its promotion. Furthermore, we have reported the evidence of a peculiar enrichment of HBsAg immune-escape mutations in the setting of HBV reactivation [6,7]. Indeed, immune-escape mutations can affect the structure and the antigenicity of HBsAg, thus contributing to HBV reuptake in the setting of a suboptimal immune system control [8].

Some specific immune-escape mutations can introduce additional N-linked glycosylation sites (Asn-X-Ser/Thr, where X is any amino acid except proline) in HBsAg. These mutations can determine the acquisition of a carbohydrate shield, thus masking the epitope(s) targeted by antibodies.

It is known that wild type (*wt*) HBsAg contains a single N-linked glycosylation site at N146 [9], however previous studies have suggested that some mutational patterns associated with HBsAg hyper-glycosylation are responsible for an altered antigenicity of HBV envelope protein, while their role in modulating viral replication ability and HBsAg secretion capacity is still controversial [10].

In this light, the main aim of this study is to investigate N-linked glycosylation patterns of HBsAg in the setting of immunosuppression-driven HBV reactivation in vivo and to evaluate their impact on HBV viral replication and HBsAg antigenic properties in vitro.

## 2. Materials and Methods

### 2.1. Study Population

This study included 47 patients developing immunosuppression-driven HBV reactivation. All patients were HBsAg-negative and positive to antibodies against HBV core antigen (HBcAg) at baseline-screening and experienced the reappearance of serum HBV-DNA (≥10 IU/mL), during or after the administration of an immunosuppressive-therapy (anti-cancer drugs and corticosteroids). All patients were infected with the HBV D genotype, according to the results of HBsAg population-based sequencing and the following phylogenetic analysis by the Tajima-Nei model (MEGA6.1).

Approval by the Ethics Committee was deemed unnecessary because, under Italian law, biomedical research is subjected to previous approval by ethics committees only in the hypothesis of clinical trials on medicinal products for clinical use (art. 6 and art. 9, leg. decree 211/2003). The research was conducted on data previously anonymized, according to the requirements set by the Italian Data Protection Code (leg. decree 101/2018).

### 2.2. HBsAg Population-Based Sequencing and Phylogenetic Analysis

The sequencing of the genomic region encoding the full-length HBsAg was performed on plasma samples, following a home-made protocol, as previously reported [6] (Supplementary Materials [SM]). HBV-DNA sequences obtained by population-based sequencing were used to infer protein sequences.

HBV genotype was determined by constructing phylogenetic tree using the neighbor-joining (NJ) method [11]. Distances were calculated using MEGA 6.1 based on the Kimura-2 parameter (K2P) model [12]. The reliability of the branching orders was assessed by bootstrap analysis of 1000 replicates.

### 2.3. Identification of Additional N-Linked Glycosylation Sites in HBsAg

The full-length HBsAg sequences were submitted to the “*N*-Glycosite” algorithm at: http://www.hiv.lanl.gov/. This algorithm identifies the presence of the signal motif for N-linked glycosylation in a given amino acid (aa) sequence. The signal motif has to begin with an asparagine (N) followed by any aa except Proline and the third aa residue has to be either a threonine (T) or a serine (S).

The following mutations associated with additional N-linked glycosylation sites were identified: 114N-ins, T115N, T117N, T123N, S113N+T131N+M133T.

### 2.4. Cell Cultures

Huh7 cells were grown in a 37 °C humidified atmosphere containing 5% CO2, using Dulbecco’s modified Eagle’s medium (DMEM) (Life Technologies, Inc., Gaithersburg, MD, USA) supplemented with 10% fetal bovine heat-inactivated serum and with 100 U/mL penicillin + 100 µg/mL streptomycin + 2 mM L-glutamine.

### 2.5. Preparation of Full-Length HBV DNA Genomes for Transient Transfection

The transfection of linear full-length HBV DNA genomes [13] recapitulates the complete post-entry transcription/replication viral cycle of HBV, allowing in particular to investigate pre-genomic RNA and sub-genomic RNA transcription from a cccDNA-like chromatinized viral mini-chromosome [14] that is functionally undistinguishable from native cccDNA from HBV infected livers [14,15,16]. Monomeric linear full-length wild type (*Wt*) HBV genotype D (subtype AYW) genomes were released by EcoRI (New England Biolabs, Inc., Ipswich, MA, USA) digestion from the recombinant plasmid pFC80 HBV.D.EcoRI that contains 4 head-to-tail 1.0× length HBV genomes (a gift of F. Chisari and P Tiollais). The 3.2-kb fragments were recovered by gel purification using the QIAquick gel extraction kit (Qiagen, Hilden, Germany) and inserted into a Topocloning vector (Invitrogen, Carlsbad, California, USA) to generate the Topo.HBV.*WT*.D plasmid. The newly identified mutations associated with additional N-linked glycosylation sites (114N-ins, T115N, T117N, T123N, S113N+T131N+M133T) were introduced into the Topo.HBV.*WT*.D plasmid by site-directed mutagenesis. *Wt* and mutant 1.0× length monomers were excised from the Topo.HBV.*WT*.D plasmid using EcoRI purified as described above and 500 ng of DNA were transfected into Huh7 cells using the TransIT-2020 Transfection Reagent (Mirus Bio LLC, Madison, Wisconsin, USA), according to the manufacturer’s instructions. All transfections included 0.1 μg of green fluorescence protein expression vector (GFP) to assess transfection efficiency. Both cell fractions and culture supernatants were harvested at 72 h post-transfection to quantify different HBV markers (see below). For each mutant, at least 3 independent transfection experiments were performed and each transfection was carried out in duplicate.

### 2.6. Quantification of HBsAg and HBV-DNA in Supernatants

Cell supernatants were collected, clarified by centrifugation at 4000 g for 5 min and used to quantify HBsAg by Elecsys^®^ HBsAg II quant (Roche, Basel, Switzerland) and HBV-DNA with cobas^®^ HBV Test (Roche, Basel, Switzerland).

### 2.7. Quantification of Core-Particles associated HBV-DNA in Cell Lysates

Transfected cells were washed once with ice-cold phosphate buffered saline (PBS) and lysed in 10 mM Tris-HCl, pH 7.4, 1 mM EDTA, 50 mM NaCl, and 1% NP-40 (lysis buffer A). Nuclei were pelleted by centrifugation for 5 min at 10,000 g (nuclear fraction) and the cytoplasmic fraction (containing the core particles associated DNA) was adjusted to 0.1 mM MgCl2 and treated with 0.1 mg/mL of DNase I for 30 min at 37 °C. The reaction was stopped by adding EDTA to a final concentration of 1 mM. Viral core particles were then precipitated in 0.8 M NaCl, 8% polyethylene glycol solution at 4 °C for 1 h. Core particles were then concentrated by centrifugation (10 min at 10,000 g) and were re-suspended in 10 mM Tris-HCl, 100 mM NaCl, 1 mM EDTA, 1% SDS, and 0.5 mg/mL proteinase K and incubated for two hours at 56 °C. Viral DNA released from lysed core particles was extracted with phenol-chloroform method and quantified by real time PCR using Light Cycler instrument (Roche, Inc). Nuclear fractions were used for the quantification of the ß-globin housekeeping gene. Pelleted nuclei were resuspended in lysis buffer B (10 mM Tris-HCl, 10 mM EDTA, 150 mM NaCl, 0.5% SDS, and 0.5 mg/mL proteinase K), and incubated overnight at 37 °C. Nucleic acids were purified by phenol/chloroform (1:1) extraction and ethanol precipitation. All PCR reactions were performed in 20 μL volume containing 3 mM MgCl2, 0.5 μM forward and reverse primers, 0.2 μM of 3′-fluorescein (FL)-labeled probe, and 0.2 μM of 5′-Red640 (R640)-labeled probe. The following HBV primers and probes were used for core particles associated HBV DNA quantification in the cytoplasmic fractions: forward 5′-CTCGTGGTGGACTTCTCTC-3′, reverse 5′-CAGCAGGATGAAGAGGAA-3′ and the specific FRET hybridization probes: 5′-CACTCACCAACCTCCTGTCCTCCAA-FL-3′, Red640-5′-TGTCCTGGTTATCGCTGGATGTG TCT-3′. HBV-DNA complete genomes from Clonit Srl (cat.no.05960116) were used for the standard curve. The following primers and probes were used for ß-globin normalization in the nuclear fractions: forward 5′-ACACAACTGTGTTCACTAGC-3′, reverse 5′-CAACTTCATCCACGTTC ACC-3′, and the specific FRET hybridization probes 5′-CAAACAGACACCATGGTGCAC CTGACTCCTGAGGA-FL-3′, Red640-5′-AAGTCTGCCGTTACTGCCCTGTGGGGCAA-3′. Amplifications were performed as follows: 95 °C for 5 min followed by 45 cycles at 95 °C for 10 s, 57 °C for 10 s, and 72 °C for 20 s.

### 2.8. Quantification of Pre-Genomic HBV-RNA in Cell Lysates

Total RNA was extracted from Huh7 cells by using the TRIzol reagent (Invitrogen, Carlsbad, California, USA) as recommended by the manufacturer. The RNA samples were treated with RQ1 RNase-Free DNase (Promega Inc., Madison, Wisconsin, USA) for 60 min at 37 °C and stored until used. RNA quality and quantity were monitored by ethidium-bromide staining and by UV absorbance. For precore/pgRNA analysis, 2 μg of DNase-treated RNA was reverse transcribed and amplified by the ThermoScript RT-PCR System (Invitrogen, Carlsbad, California, USA). Then, 2 μL of each cDNA was quantified by real-time PCR analysis using the following core/pgRNA-specific primers and probes: forward primer, 5′-GCCTTAGAGTCTCCTGAGCA-3′, reverse primer, 5′-GAGGGAGTTCTTCTTCTAGG-3′, FRET hybridization probes, 5′–AGTGTGGATTCGCACTCCTCCAGC-FL-3′, and Red640-5′ATAGACCACCAA ATGCCCCTATCTTATCAAC-3′. Control PCR reactions using DNase-treated RNA aliquots not subjected to reverse transcription were included in all experiments. The h-GAPDH house-keeping gene Light Cycler set (Roche Diagnostics, Basel, Switzerland) was used to normalize the RNA samples.

### 2.9. Impact of Mutations on HBsAg Antigenic Properties or Secretion

In order to better unravel the role of the identified N-linked glycosylation sites in affecting HBsAg secretion or antigenic properties, a plasmid encoding the HBsAg linked to a streptavidin-tag (version II, IBA Lifesciences, Göttingen, Germany) (strep-tag) was used to transfect the Huh7 cells. The strep-tag is upstream the gene encoding HBsAg, thus resulting at the N-terminus of the corresponding protein.

After 72 h post transfection, cell supernatants were collected, clarified by centrifugation at 4000 g for 5 min, and used for the quantification of strep-tag HBsAg. In particular, the amount of strep-tagged HBsAg released in culture supernatants was quantified by using two assays targeting the major hydrophilic region of HBsAg (Monolisa™ HBs Ag ULTRA [Bio-Rad Laboratories, Hercules, CA, USA] and Elecsys^®^ HBsAg II quant [Roche, Basel, Switzerland]) and by using a specifically-designed ELISA capable to recognize the Strep-tag portion linked to the HBsAg (defined hereafter as Strep-tag ELISA). Differently from the commonly used HBsAg assays, the Strep-tag based ELISA is not influenced by HBsAg modifications, giving the advantage to discriminate between a reduction of HBsAg recognition (due to an altered HBsAg antigenic properties) and a decrease in HBsAg secretion.

For each mutant, at least 3 independent transfection experiments were performed, each carried out in duplicate.

### 2.10. Tunicamycin Treatment

In order to confirm if the additional N-linked glycosylation sites identified by analyzing HBsAg sequences were actually glycosylated, a treatment with tunicamycin, an inhibitor of N-linked glycosylation was performed. Huh7 cells were transfected by using the plasmid encoding wt or mutated strep-tag HBsAg and treated with tunicamycin (Sigma-Aldrich, St. Louis, Missouri, USA). Tunicamycin was dissolved in DMSO and added to cells at 6 h after transfection at a final concentration of 1 µg/mL. An equivalent concentration of DMSO was included in control cultures. The cultures were maintained for a further 48 h until cells were assayed.

### 2.11. Immunoblotting

Cells, treated and not treated with tunicamycin, were lysed with RIPA buffer (50 mM Tris-HCL ph8, 150 mM NaCl, 1% NP40, 0.5% sodium deoxycholate, 0.1% SDS). Protein concentrations were determined using the Bradford Protein Assay Reagent (Bio-Rad Laboratories, Hercules, USA).

Protein lysates were separated by SDS-PAGE gels, transferred to nitrocellulose membranes, and incubated with the antibody StrepMAB-Classic HRP conjugate (IBA Lifesciences, Gottingen, Germany), targeting the strep-tag portion of strep-tagged HBsAg.

### 2.12. Statistical Analysis

Association between the presence of additional N-linked glycosylation sites and HBsAg negativity at HBV-reactivation was assessed by Fisher exact test. Statistically significant differences between wt and mutated HBsAg in in vitro experiments were assessed by 2-tailed Student’s t test. All statistical analyses were performed by using IBM SPSS (IBM Corp. Released 2013., IBM SPSS Statistics for Windows, Version 23.0., Armonk, NY, USA).

## 3. Results

### 3.1. Study Population

This study included 47 consecutive non-selected patients experiencing immunosuppression-driven HBV reactivation (Table 1). Patients were mainly male (70.2%) with a median age of 64 (58–73) years and all of them harboured HBV genotype D at HBV reactivation. Most patients were receiving an immune-suppressive therapy for an onco-hematological disease (87.2%), followed by kidney transplantation (6.4%), chronic inflammatory disease (4.3%), and solid tumor (2.1%) (Table 1).

At baseline screening of HBV markers before starting immunosuppressive-therapy, all patients were negative to HBsAg (47/47, 100%). Among them, 53.2% was positive only to the antibodies against HBcAg (a profile defined as isolated anti-HBc), 31.9% positive to antibodies against HBcAg and HBsAg, 10.6% positive only to antibodies against HBsAg (though not vaccinated), and 4.3% was negative to all HBV serological markers, a profile compatible to the so-called sero-negative occult HBV-infection (Table 1).

At HBV-reactivation, median (IQR) serum HBV-DNA was 6.0 (3.6–7.5) logIU/mL and median (IQR) HBsAg levels were 6840 (115–15,037) IU/mL. Most patients experienced a biochemical reactivation with elevation of transaminases [median (IQR) ALT and AST: 474 (118–841) and 240 (100–495) U/L, respectively].

Notably, at HBV-reactivation, 23.4% (11/47) of patients remained HBsAg-negative despite a detectable serum HBV-DNA (median [IQR] HBV-DNA: 3.7 [3.3–4.3] log IU/mL).

By analyzing HBsAg mutational profiles, HBsAg negativity at HBV reactivation (thus, despite ongoing viral replication) was significantly correlated with the presence of at least one additional *N*-glycosylation site in HBsAg MHR. Indeed, ≥1 additional glycosylation site in MHR is found in 45.5% of patients with HBsAg negativity at HBV-reactivation and in none of the patients with HBsAg positivity at HBV reactivation (*p* < 0.001). No other HBV genetic characteristics resulted to be correlated with HBsAg negativity at HBV reactivation.

In particular, the additional *N*-Glycosylation sites in HBsAg MHR derive from a single aa substitution in 3 patients (T115N, T117N, and T123N, respectively) and from the insertion of 114N-ins in a single patient. In the remaining patient, the pattern of mutations S113N+T131N+M133T was observed, resulting in the introduction of 2 different glycosylation sites. All these mutations determine the acquisition of additional N-X-S/T motifs in HBsAg beyond the classical motif at position 146–148.

Among the 5 patients with additional *N*-glycosylation sites, 4 remained HBsAg negative during follow-up (>12 months) after starting antiviral treatment. The remaining patient died 2 months after HBV reactivation showing HBsAg-negativity in the sample closest to death.

### 3.2. Impact of the Additional N-glycosylation Sites in HBsAg MHR on HBsAg Release and on Viral Replication by In Vitro Experiments

The first step of in vitro experiments was to investigate the impact of these additional *N*-glycosylation sites on HBsAg secretion in cell cultures and on HBV replication.

**Figure 1:**
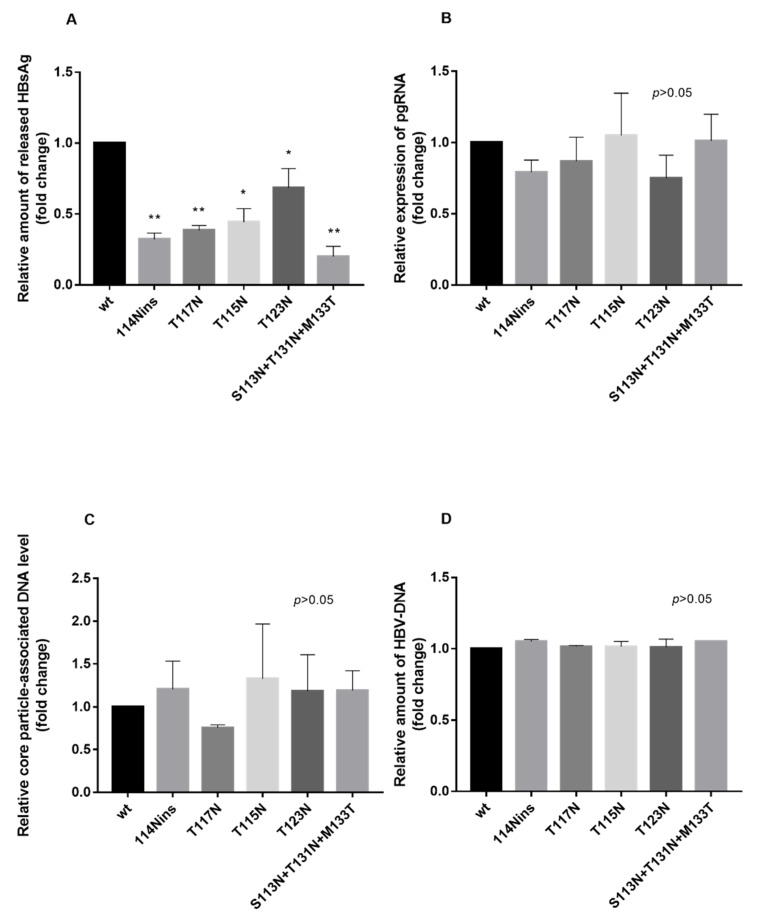
Impact of the additional *N*-glycosylation site in HBsAg MHR on viral replication by in vitro experiments. Huh7 cells were transfected with a wt and mutated Hepatitis B virus (HBV) full-genome plasmid (genotype D). 72 h post transfection the amount of HBsAg in supernatants (**A**), intracellular pre-genomic HBV-RNA (**B**) and core-associated HBV-DNA (**C**) as well as HBV-DNA in supernatants (**D**) were measured. Values are the mean (±SD) of at least three independent experiments each carried out in duplicate and expressed as relative amount compared to wt. HBsAg and HBV-DNA in supernatants were measured by Elecsys^®^ HBsAg II quant (Roche, Basel, Switzerland) and ROCHE COBAS TaqMan HBV test;v2.0^®^, respectively, while intracellular pre-genomic HBV-RNA and core-associated HBV-DNA by home-made real time PCR. Statistically significant differences between each mutant and wt in the quantification of the abovementioned HBV parameters were assessed by Student’s *t*-test. * *p* < 0.05, ** *p* < 0.01.

The presence of ≥1 additional N-linked glycosylation site determined a significant decrease in the HBsAg amount released in supernatant compared to wt. In particular, the N-linked glycosylation sites 114Nins, T117N, T115N, and T123N showed a 68%, 62%, 56%, and 45% decrease in extracellular HBsAg amount (*p*-values ranging from 0.05 to 0.01), respectively (Figure 1A). Notably, for the triple mutant S113N+T131N+M133T, an 80% decrease in extracellular HBsAg amount was observed (*p* < 0.01).

Conversely, the presence of N-linked glycosylation sites did not significantly affect the amount of intracellular pre-genomic HBV-RNA and core-associated HBV-DNA, as well as of extracellular HBV-DNA compared to wt (*p* > 0.05) (Figure 1B–D). These results suggest that the introduction of the identified additional N-linked glycosylation sites did not alter the efficiency of HBV replication.

### 3.3. Role of Additional N-linked Glycosylation Sites on HBsAg Antigenic Properties

The next step of this study was to evaluate whether the decreased HBsAg amount observed in the presence of additional N-linked glycosylation sites can be related to a defect in HBsAg secretion or to a modification of HBsAg antigenic properties. In order to unravel this issue, a plasmid encoding the strep-tagged HBsAg, carrying the wild-type sequence or the above-mentioned N-linked glycosylation sites, was used to transfect Huh7 cells. The amount of strep-tagged HBsAg released in supernatants was quantified by using two ELISAs targeting the MHR of HBsAg and an ELISA targeting the streptavidin tag.

**Figure 2:**
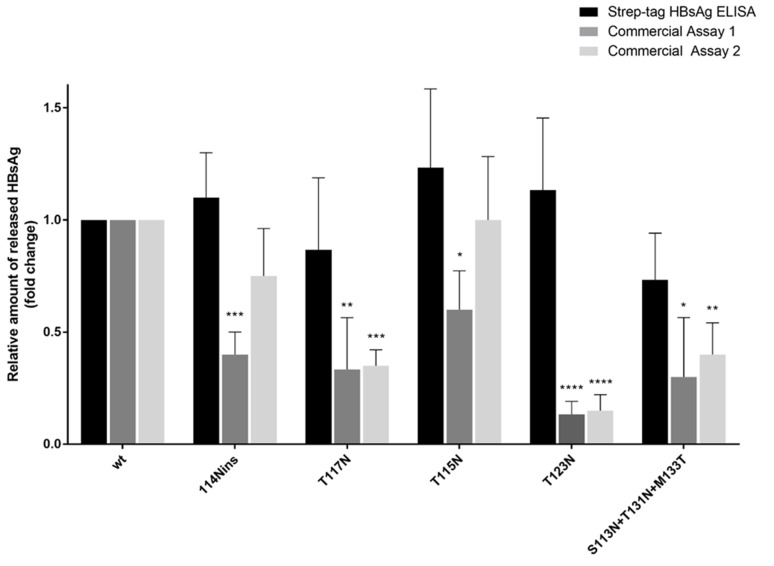
Impact of *N*-glycosylation on HBsAg antigenicity. Quantification of strep-tagged HBsAg released in the supernatants of Huh7 cell cultures by different assays is shown. Values are the mean (±SD) of three independent experiments each carried out in duplicate and expressed as relative amount compared to wt. The black bars show the Strep-tagged HBsAg quantified by an ELISA using antibodies targeting Strep-tag portion. The dark and light grey bars report the Strep-tagged HBsAg quantified with commercial assays (thus, using antibodies targeting the major hydrophilic region of HBsAg). Statistically significant differences in the amount of extracellular Strep-tagged HBsAg between each mutant and wt were assessed by Student’s *t*-test. * *p* < 0.05, ** *p* < 0.01, *** *p* < 0.001, **** *p* < 0.0001

The N-linked glycosylation sites determined a decrease in the quantification of the strep-tagged HBsAg when the two ELISAs targeting the MHR of HBsAg were used (Figure 2). In particular, all the N-linked glycosylation sites determined a statistically significant decrease (ranging from 40% to 90%) in extracellular strep-tagged HBsAg for the former ELISA. For the latter ELISA, a statistically significant decrease in the amount of extracellular strep-tagged HBsAg (ranging from 60% to 90%) was observed for 3/5 of the analyzed N-linked glycosylation sites (Figure 2).

Conversely, by using the ELISA targeting the strep-tag, the additional N-linked glycosylation sites did not affect the quantification of the strep-tagged HBsAg, that was not significantly different to that observed for wt (Figure 2). These results confirm that the N-linked glycosylation sites hamper HBsAg recognition and quantification by diagnostic antibodies without affecting HBsAg release.

### 3.4. Impact of HBsAg Mutations on N-Linked Glycosylation

**Figure 3:**
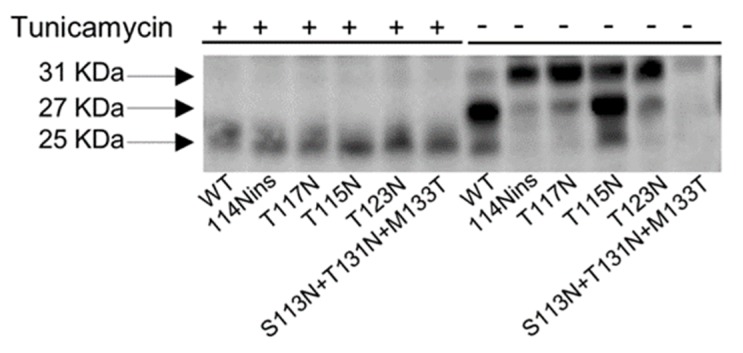
Immunoblotting of Strep-tagged HBsAg in presence of (referred as “+” in the figure) and in absence of (referred as “-” in the figure) tunicamycin treatment. Huh7 cells were transfected with a plasmid encoding wt and mutated strep-tagged HBsAg. After 6 h, the glycosylation inhibitor tunicamycin was added at concentration of 1 μg/mL. After 48 h, cell lysates were subjected to SDS-polyacrylamide gel electrophoresis and detected with antibodies targeting the Strep-tag portion of strep-tagged HBsAg. The 25 KDa band corresponds to the un-glycosylated form of strep-tagged HBsAg, the 28 KDa band to the HBsAg glycosylated at the classical N-linked glycosylation site at position 146, while the 31 KDa band corresponds to the HBsAg form glycosylated at the position 146 and at the novel identified N-linked glycosylation sites.

In order to confirm if the N-X-S/T motifs are actually used for N-linked glycosylation, cells transfected with wt and mutated strep-tagged HBsAg were treated with the glycosylation inhibitor tunicamycin. In absence of tunicamycin, two bands were detected in wt HBsAg as expected: the former corresponding to the unglycosylated HBsAg and the latter to the HBsAg isoform glycosylated at the classical N-linked glycosylation position 146–148. All the N-X-S/T motifs identified in this study determined the acquisition of an extra lower-mobility band, corresponding to the HBsAg isoforms glycosylated at both the classical 146–148 position and at the novel identified motif (Figure 3).

In line with these results, in presence of tunicamycin, only a single band with the lowest molecular weight was detected for both wt and for each additional N-linked glycosylation sites, supporting the abrogation of N-linked glycosylation.

Furthermore, transfection experiments were performed with the plasmids encoding *wt* and mutated strep-tagged HBsAg in presence or absence of tunicamycin treatment. Step-tagged HBsAg were measured in supernatants by using a commercial ELISA targeting the major hydrophilic region of HBsAg. HBsAg levels of mutated clones were higher in presence of tunicamycin treatment than in absence of this N-linked glycosylation inhibitor, confirming the role of *N*-glycosylation in altering HBsAg recognition (Figure S1).

## 4. Discussion

This study shows that a not negligible fraction of patients, negative to HBsAg and positive to antibodies against HBcAg (22.9%), remains HBsAg-negative at HBV reactivation despite ongoing viral replication. The analysis of HBsAg genetic characteristics reveals that HBsAg negativity was associated with the presence of additional N-linked glycosylation sites in the MHR of HBsAg.

Our result is in line with a French study showing that 18.7% of patients (negative to HBsAg and positive to antibodies against HBcAg at baseline-screening) remained HBsAg-negative at the diagnosis of HBV reactivation [17]. Similarly, an Asiatic study has shown that 11/13 immunosuppressed patients (again negative to HBsAg and positive to antibodies against HBcAg at baseline-screening) displayed HBsAg-negativity during the re-uptake of HBV replication [18]. Overall, these findings corroborate the use of serum HBV-DNA for monitoring patients at risk of HBV reactivation or the need of innovative serological assays for HBsAg quantification targeting not only MHR but also other conserved HBsAg regions.

The analysis of HBsAg mutational profiles highlights that HBsAg-negativity can be explained by the acquisition of additional N-linked glycosylation sites in the MHR of HBsAg. N-linked glycosylation sites are known to play a critical role in viral evasion from humoral responses [19,20]. Indeed, the introduction of a carbohydrate shield can mask B-cell epitopes and thus protein surface targets of antibodies. The detection of additional N-linked glycosylation sites in the setting of HBV-reactivation is in line with previous studies showing that viral strains circulating in patients with HBV reactivation are characterized by an enrichment of immune escape mutations in HBsAg MHR, which can provide a selective advantage during the progressive weakening of the immune system. This study further reinforces the role of immune-escape in mechanisms underlying immune-suppression driven HBV reactivation.

At the same time, the acquisition of N-linked glycosylation sites can pose a diagnostic issue since they can alter HBsAg recognition by antibodies used in diagnostic assays. By using an ad-hoc designed assay, we have shown that the additional N-linked glycosylation sites identified in this study affect the antigenic properties of HBsAg MHR, without altering HBsAg secretion and HBV replicative potential. This can explain the HBsAg negativity observed in patients at HBV reactivation, despite ongoing viral replication. Our results are in line with previous studies showing that HBsAg mutants with new *N*-glycosylation sites reacted weakly with antibodies against HBsAg [19]. Considering the N-linked glycosylation sites observed in our study, recent studies have shown that the mutational profile T131N+M133T reduces HBsAg antigenicity and fails to induce anti-HBs response without altering HBV replicative capacity [10,21]. Other studies have also shown the capability of T131N+M133T to rescue HBsAg secretion impaired by the vaccine escape mutation G145R [22,23].

Similarly, the detection of T123N is capable to affect HBsAg antigenic properties [24,25] and has been observed in patients with atypical serological profiles for HBV infection [26]. Furthermore, another study has shown that the N-linked glycosylation site T123N can strongly reduce virion assembly when introduced in a HBV genotype C backbone [27]. In our study, T123N did not affect HBV replicative efficiency when present in a HBV genotype D backbone. This result suggests that the impact of HBsAg mutations on HBV replication can be influenced by the genetic backbone of HBV genotype.

A recent study has highlighted that occult HBV infection is characterized by an enrichment of mutations associated with the acquisition of N-linked glycosylation sites [28]. This is in agreement with a previous study by our group, showing the associated of T115N and T123N with occult HBV infection in the setting of HBV genotype D. It is conceivable that such mutations can be generated during the establishment of occult HBV infection and remained archived in the cccDNA to re-emerge in the setting of immune-suppression driven HBV reactivation.

In conclusion, a not negligible fraction of patients, HBsAg negative at baseline screening, remained HBsAg-negative at immunosuppressive-driven HBV reactivation despite ongoing viral replication. Additional N-linked glycosylation sites can contribute to such HBsAg-negativity by altering HBsAg antigenic properties. This supports the role of *N*-glycosylation in immune-escape and the importance of HBV-DNA quantification or innovative assays for HBsAg quantification targeting not only MHR, but also other conserved HBsAg regions for a proper diagnosis of HBV-reactivation.

## Figures and Tables

**Table 1:** Patients’ characteristics.

**Patients’ Characteristics**	**HBV Reactivated Patients (*n* = 47)**
Male, *n* (%)	33 (70.2)
Italian origin, *n* (%)	43 (91.5)
Median age, years (IQR)	64 (58–73)
**Pathologies Requiring Immunosuppressive Therapy**	
Onco-hematological disease, *n* (%)	41 (87.2)
Kidney Transplantation, *n* (%)	3 (6.4)
Chronic inflammatory disease, *n* (%)	2 (4.3)
Solid tumor, *n* (%)	1 (2.1)
**Pre-Reactivation Status**	
Positive only to antibodies against HBcAg, *n* (%)	25 (53.2)
Positive to antibodies against HBsAg and HBcAg, *n* (%)	15 (31.9)
Positive only to antibodies against HBsAg, *n* (%)	5 (10.6)
Negative to all HBV serological markers, *n* (%)	2 (4.3)
**Virological and Biochemical Characteristics at HBV Reactivation**	
HBsAg positive, HBV-DNA positive, *n* (%)	36 (76.6)
HBsAg negative, HBV-DNA positive, *n* (%)	11 (23.4)
HBV Genotype-D, *n* (%)	47 (100)
Median HBV-DNA, log10 IU/mL (IQR)	6.0 (3.6–7.5)
Median quantitative HBsAg, IU/mL (IQR)	6840 (115–15037)
Median ALT, U/L (IQR)	144 (37–682)
Median AST, U/L (IQR)	120 (36–427)

Abbreviations: HBV, Hepatitis B virus; HBcAg, HBV core antigen; HBsAg, HBV surface antigen; ALT, alanine aminotransferase; AST, aspartate aminotransferase.

## Supplementary Materials

The following are available online at https://www.mdpi.com/1999-4915/12/2/251/s1. Supplementary materials and Figure S1: Strep-tag HBsAg quantification with or without tunicamycin treatment.
