# Supplementary material for: A Hyper-Glycosylation of HBV Surface Antigen Correlates with HBsAg-Negativity at Immunosuppression-Driven HBV Reactivation in Vivo and Hinders HBsAg Recognition In Vitro
Source: Viruses. 2020 Feb 23;12(2):251. doi: 10.3390/v12020251 (PMC7077195; doi:10.3390/v12020251)
Supplement: Supplementary file 1 [file viruses-12-00251-s001.zip › Supplementary_figure_1.pptx]

## Slide 1
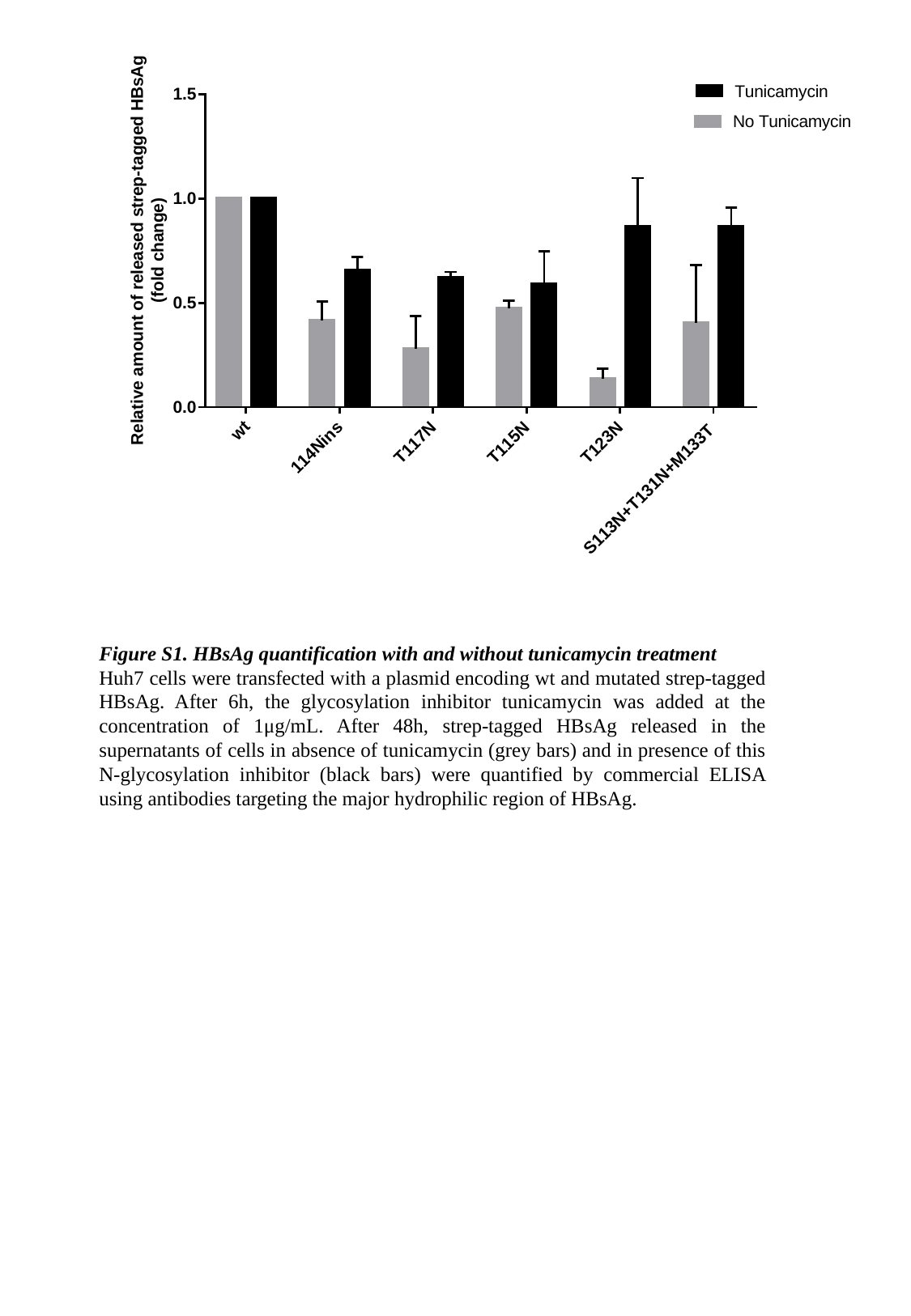

Figure S1. HBsAg quantification with and without tunicamycin treatment
Huh7 cells were transfected with a plasmid encoding wt and mutated strep-tagged HBsAg. After 6h, the glycosylation inhibitor tunicamycin was added at the concentration of 1μg/mL. After 48h, strep-tagged HBsAg released in the supernatants of cells in absence of tunicamycin (grey bars) and in presence of this N-glycosylation inhibitor (black bars) were quantified by commercial ELISA using antibodies targeting the major hydrophilic region of HBsAg.
