# Supplementary material for: A Hyper-Glycosylation of HBV Surface Antigen Correlates with HBsAg-Negativity at Immunosuppression-Driven HBV Reactivation in Vivo and Hinders HBsAg Recognition In Vitro
Source: Viruses. 2020 Feb 23;12(2):251. doi: 10.3390/v12020251 (PMC7077195; doi:10.3390/v12020251)
Supplement: Supplementary file 1 [file viruses-12-00251-s001.zip › Supplementary_materials.docx]

**Protocol for HBsAg sequencing**

HBV-DNA was extracted using a commercially available kit (QIAmp DNA blood mini-kit, Qiagen Inc., USA), and then amplified with Amplitaq-Gold polymerase using the following primer pairs: F1-5’GGTCACCATATTCTTGGGAA and R1-5’GTGGGGGTTGCGTCAGCAAA. PCR conditions were: one cycle at 93 °C for 12 min, 40 cycles (94 °C 50 s, 57 °C 50 s, 72 °C 1 min and 30 s), and a final cycle at 72 °C for 10 min. For samples with serum low HBV-DNA, 2 additional heminested-PCR were performed, starting from the same first amplicon: heminested-1 used the following primer pairs (F1-5’GGTCACCATATTCTTGGGAA and R2-GAGGACAAACGGGCAACATACCTT and heminested-2 used F2-GTTGACAAGAATCCTCACAATA and R1-5’GTGGGGGTTGCGTCAGCAAA. Both hemi-nested-PCRs conditions were: one cycle at 93 °C for 12 min, 35 cycles (94 °C 50 s, 56 °C 50 s, 72 °C 1 min), and a final cycle at 72 °C for 10 min. PCR-products were purified and sequenced by using eight different overlapping sequence-specific primers, a BigDye terminator v. 3.1 cycle sequencing kit (Applied-Biosystems FosterCity, CA) and an automated sequencer (Genetic Analyzer 3130XL). The sequences were analyzed using SeqScape-v.2.5 software. The quality endpoint for each individual sequence was ensured by a coverage of the S gene sequence by at least two segments. Sequences having a mixture of wild-type and mutant residues at single positions were considered to have the mutant(s) at that position.
